# Hemoglobin, Lead Exposure, and Intelligence Quotient: Effect Modification by the *DRD2* Taq IA Polymorphism

**DOI:** 10.1289/ehp.0901878

**Published:** 2010-09-24

**Authors:** Ananya Roy, Howard Hu, David C. Bellinger, Bhramar Mukherjee, Rama Modali, Khaja Nasaruddin, Joel Schwartz, Robert O. Wright, Adrienne S. Ettinger, Kavitha Palaniapan, Kalpana Balakrishnan

**Affiliations:** 1 Environmental and Occupational Health Sciences Institute, University of Medicine and Dentistry of New Jersey, Piscataway, New Jersey, USA; 2 Department of Environmental Health Sciences, University of Michigan School of Public Health, Ann Arbor, Michigan, USA; 3 Department of Environmental Health, Harvard School of Public Health, Boston, Massachusetts, USA; 4 Department of Neurology, Children’s Hospital Boston, Boston, Massachusetts, USA; 5 Department of Biostatistics, University of Michigan School of Public Health, Ann Arbor, Michigan, USA; 6 BioServe Biotechnologies, Hyderabad, India; 7 Channing Laboratory, Brigham and Women’s Hospital, Boston, Massachusetts, USA; 8 Department of Medicine, Children’s Hospital Boston, Boston, Massachusetts, USA; 9 Department of Environmental Health Engineering, Sri Ramachandra Medical College and Research Institute, Chennai, Tamil Nadu, India

**Keywords:** blood lead, children, dopamine, hemoglobin, India, intelligence quotient

## Abstract

**Background:**

Anemia and lead exposure remain significant public health issues in many parts of the world, often occurring together. Animal studies suggest that the dopamine D2 receptor (DRD2) mediates the effects of both lead and iron on cognition and behavior.

**Objective:**

We tested the hypothesis that the *DRD2* Taq IA polymorphism modifies the effects of lead and hemoglobin on intelligence quotient (IQ) among children.

**Methods:**

Blood lead and hemoglobin were assessed in 717 children 3–7 years of age attending 12 schools in Chennai, India. IQ was determined using the Binet-Kamat scales of intelligence. Genotyping for the *DRD2* polymorphism was carried out using a MassARRAY iPLEX platform. Stratified analyses and interaction models, using generalized estimating equations (GEEs), were used to explore interactions between lead and hemoglobin, and *DRD2* Taq IA categories [homozygous variant (A1) vs. presence of wild-type allele (A2)].

**Results:**

After we controlled for potential confounders, a one-unit increase in log blood lead was associated with a decrease of 9 IQ points [95% confidence interval (CI), −18.08 to −0.16] in the homozygous-variant children (*n* = 73) compared with a decrease of 4 IQ points (95% CI, −7.21 to −0.69) among those with the wild-type allele (*n* = 644). Higher hemoglobin levels were associated with higher IQ in the children who carried the wild-type allele *DRD2*, but in children homozygous for the variant allele, an increase of 1 g/dL hemoglobin was associated with a decrease in 1.82 points of IQ (95% CI, −5.28 to 1.64; interaction term *p*-value = 0.02).

**Conclusion:**

The results of this study suggest that the *DRD2* Taq IA polymorphism disrupts the protective effect of hemoglobin on cognition and may increase the susceptibility to the deficits in IQ due to lead exposure.

Iron-deficiency anemia (IDA) and lead exposure remain significant public health problems. It is estimated that the prevalence of IDA is around 46–66% in developing countries (Stoltzfus et al. 2007). In India, 74% of urban children < 3 years of age are anemic [International Institute for Population Sciences (IIPS) and Macro International 2007].

Although a number of countries have recently phased out lead from gasoline, lead exposure at the population level in some locations, particularly in developing countries, remains high. Mean blood lead levels are reported to be 13 μg/dL in China (Ye et al. 2007), 15 μg/dL in urban populations in Bangladesh (Kaiser et al. 2001; Wasserman et al. 2007), and 11 μg/dL among urban children in India (Roy et al. 2009a). In contrast, developed countries, such as the United States, report mean blood lead levels of 1.9 μg/dL among children (Jones et al. 2009).

A large body of literature documents the adverse effects of lead exposure and iron deficiency on cognitive function among children (Canfield et al. 2003; Lanphear et al. 2005; Lozoff and Georgieff 2006; McCann and Ames 2007). The large variability in effect estimates, between and within study populations, could reflect measurement error and residual confounding, along with between-person and between-population variation in susceptibility, due to factors such as genetic polymorphisms. Currently, little is known about genetic factors that may modify the effects of lead and iron on children’s neurocognition.

Information from experimental and observational research suggests that genetic polymorphisms within the dopamine neurotransmitter pathway could influence neurological susceptibility to both lead and IDA. It is well documented that both iron and lead levels are associated with changes in the dopamine system, which in turn modulate behavior and cognition (Beard 2007; Beard et al. 2006; Brockel and Cory-Slechta 1999; Cory-Slechta 2003; Cory-Slechta et al. 1998, 2002; Unger et al. 2007). Animal studies suggest that autoregulation of the dopamine system via the dopamine receptor D2 (DRD2) plays an important role in the neurobiological effect of lead and iron (Beard and Connor 2003; Cory-Slechta 1995; Erikson et al. 2001; Lozoff et al. 2006).

A recent study by Froehlich et al. (2007) suggests that the effect of lead on executive function among children is modified by dopamine D4 receptor genotype, indicating that polymorphisms in the dopamine system may be important sources of variability in the association between lead and childhood cognition.

The human *DRD2* gene is located at 11q22-q23. It is associated with TaqI restriction-fragment-length polymorphism (Taq IA polymorphism, rs1800497), creating A1 and A2 alleles (Wong et al. 2000). Although Taq IA is in the ankyrin repeat and protein kinase domain containing protein 1 (*ANKK1*) gene, which lies 10,541 bp downstream of the termination codon of the *DRD2* gene, this polymorphism is in strong linkage disequilibrium with two other functional *DRD2* single-nucleotide polymorphisms (SNPs; rs2283265 and rs1076560) that affect DRD2 receptor isoform expression (Ponce et al. 2009). Studies have shown that healthy individuals who are A1 allele carriers have 10–30% lower DRD2 density and binding in the brain (Jonsson et al. 1999; Noble et al. 1991; Pohjalainen et al. 1998; Thompson et al. 1997). In addition, it has been suggested that the A1 allele may diminish dopaminergic activity in the central nervous system, resulting in prolonged P300 latency, which is an event-related potential recorded by electroencephalographic techniques and associated with cognitive processing (Berman et al. 2006) or reduced brain glucose metabolism (Noble et al. 1997). In addition, the A1 allele has been associated with a multitude of psychiatric disorders (Pacit et al. 2010; Ponce et al. 2009), as well as decreased learning and verbal creativity (Jocham et al. 2009; Reuter et al. 2005, 2006), indicating that this particular polymorphism may affect dopamine mediated behavior and cognition.

Thus, we hypothesized that the effect of hemoglobin (as a measure of iron) and lead on cognition [intelligence quotient (IQ)] among children is modified by the *DRD2* Taq IA polymorphism.

## Materials and Methods

### Study population and design

The study population has been described previously (Roy et al. 2009a, 2009b). In brief, four different industrial and traffic zones were selected to represent the range of exposure in Chennai, India. Three schools from each of the four zones were randomly selected (12 schools total), and all 814 children 3–7 years of age from lower and upper kindergartens and first grade were eligible to be participants in this study.

The parents of the children were invited to group discussions with the research team at the schools, and informed consent was acquired from the primary caregiver of each child. We received informed consent from the caregivers of all 814 children, but because some children were absent or sick on the day of blood draw, only 756 (92.8%) participated in the study.

The study was cross-sectional, with cognitive testing and blood collection for lead and genotype analyses done on the same day. A trained interviewer orally administered a questionnaire in Tamil (the regional language) to the primary caregiver of the child to collect information on other covariates.

This collaborative study on lead genetics and neurotoxicity was conducted by the Harvard School of Public Health (HSPH; Boston, MA, USA) and Sri Ramachandra Medical College and Research Institute (SRMC; Chennai, India). The study was approved by institutional review boards at HSPH, University of Michigan School of Public Health, SRMC, and the Indian Council for Medical Research (New Delhi, India) and complied with all federal U.S. and Indian regulations and laws regarding research in human subjects.

### Assessment of cognitive function

The IQ of each child (*n* = 756) was derived using the standardized Tamil-translated Binet-Kamat Scales of Intelligence (Kamat 1967), which was administered in a separate quiet room on the school premises by trained research personnel who were blinded to all aspects of the children’s lead exposure and developmental history. This amended and validated version of the 1934 Stanford-Binet Scales of Intelligence is a culture-specific scale that uses Indian pictorial scenes and concepts. The test is designed to measure aptitude and intellectual capacity through assessment of verbal reasoning, comprehension, quantitative reasoning, vocabulary, abstract reasoning, similarities, differences, visual reasoning, and short-term memory. The child’s mental age (in months) was calculated based on his or her performance on the Binet-Kamath IQ Test. The mental age was then divided by the chronological age (in months) and multiplied by 100 to obtain the child’s IQ.

### Assessment of blood lead and hemoglobin

Whole blood (10 mL) was collected from the cubital vein of each child into lead-free tubes (Becton-Dickinson blue-top tubes; Becton-Dickinson, Franklin Lakes, NJ USA) by a trained phlebotomist during a school day.

Blood lead was measured at SRMC, using a LeadCare Analyzer instrument (ESA Laboratories, Chelmsford, MA, USA), which is a well-validated field instrument with limit of detection of 1.4 μg/dL blood lead, range of 1.4–65 μg/dL, and resolution of 0.1 μg/dL (Shannon and Rifai 1997). Duplicates and ESA company controls were run every 20 samples and with every new batch and kit lot. The instrument was calibrated every time a new test kit batch or control batch was used.

Blood hemoglobin concentrations (grams per deciliter) were measured using standard clinical methods on a BC-3000 Plus Auto Hematology Analyzer (MINDRAY; Bio-Medical Electronics Co., Ltd., Shenzhen, China) at the Pathology and Hematology Laboratory in SRMC. The auto analyzer has a range of 1–25 g/dL for hemoglobin. Hemoglobin levels were missing, due to laboratory error, for three study participants.

### Genotyping

DNA was extracted from an aliquot of the collected blood of each child and analyzed for the *DRD2* Taq IA (A1, A2) allele SNP (rs1800497; SNP500Cancer ID ANKK1-01). The sequence, including 600 bp upstream and downstream of the SNP, was ascertained from Genewindow (http://genewindow.nci.nih.gov/Welcome). All polymerase chain reaction (PCR) and extension primers were synthesized at BioServe Biotechnologies, Ltd. (Beltsville, MD, USA). Genotyping was performed by BioServe at their Hyderabad, India, facility using a MassARRAY iPLEX platform (Sequenom, San Diego, CA, USA), which is a PCR process and mass spectrometry–based system.

To ensure that the samples were processed correctly and without external contamination, quality control checks involved four positive controls for each assay and four negative controls (no template controls) per plate. The positive controls were 24 DNA samples, in duplicate from the National Institutes of Health Polymorphism Discovery Panel (Collins et al. 1999). They were selected to cover all the possible genotypes available for the multiplex assay panel developed. The peaks for individual assays were checked manually in addition to Sequenom’s SpectroTYPER software automated detection.

The full data set was anonymized after genotyping to protect the confidentiality of our study participants and to conform to current institutional review board policies.

### Statistical analysis

The genotype frequencies were calculated, and the observed frequencies were tested against expected frequencies according to Hardy–Weinberg equilibrium principles using the chi-square statistic.

Univariate distributions of all variables of interest were explored across *DRD2* polymorphism type. Blood lead levels were natural log transformed to maintain a normal distribution. Bivariate analysis of variance tests were conducted to ascertain if the distributions of covariates differed among the *DRD2* Taq IA genotypes. In all multivariate analyses, outliers in lead (*n* = 7) and IQ (*n* = 1) identified using the generalized extreme studentized deviate method were removed (Rosner 1983).

We built multivariate models using ordinary least squares regression to estimate associations between IQ and blood lead and/or hemoglobin. Covariates [age + age^2^ (months), sex, midarm circumference (centimeters), average monthly family income (< 2,000, 2,000–4,000, 4,000–6,500, > 6,500 Indian rupees), and family size] were retained in the regression model if they changed the *R*^2^ and/or main effect estimate by > 10%. Parental education (both mother and father: illiterate/primary school, middle school, high school, college) was included in the multivariate model in order to maintain consistency with other studies that evaluated the impact of lead and hemoglobin on cognition.

Because the study used a multilevel sampling system, correlation among levels of sampling (classroom, school, and zone) were calculated using intraclass correlations in a random effects model. Because the distributions of the residuals were not strictly normal, we used generalized estimating equations (GEEs), which use quasi-likelihood methods to model the marginal associations after accounting for clustering at the classroom and school level (Qaqish and Liang 1992).

Our first step was to create a multivariate main effects model to estimate the effects of lead, hemoglobin, and *DRD2* Taq IA genotypes [categorized as A1/A1, A1/A2, or A2/A2 (referent)] on IQ, adjusted for age (age and age^2^, in months), sex, midarm circumference (centimeters), average monthly family income (four categories), parental education (mother and father: illiterate/primary school, middle school, high school, college), and family size (model 1). Next we estimated effects of lead and hemoglobin on IQ according to genotype (three categories) using stratified models adjusted for the covariates above (model 2).

We then combined the *DRD2* Taq IA allele categories into two categories (A1/A1 vs. A1/A2 and A2/A2 combined). In model 3, we estimated the effects of lead and hemoglobin on IQ stratified across these categories, and in model 4 we evaluated the interaction between lead and *DRD2* Taq IA polymorphism and hemoglobin and *DRD2* Taq IA polymorphism. The interaction terms (hemoglobin × *DRD2* Taq IA category and lead × *DRD2* Taq IA category) were modeled first in separate models, while adjusting for the other exposure and then jointly, to assess joint interactions, after controlling for all other potential confounders. A three-way interaction (hemoglobin × lead × *DRD2* Taq IA category) was not assessed because no interaction between lead and hemoglobin was seen.

To assess the dose–response relationship between hemoglobin/lead and IQ within the *DRD2* Taq IA genotype categories, penalized splines in generalized additive mixed models, accounting for cluster design, were used (Wood 2006). When nonlinearity was present, the penalized spline model was compared with the linear model using Akaike’s information criterion and generalized cross-validation.

The α-level for all statistical tests of significance was set at 5%. Statistical analyses were performed using SAS for Windows, version 9.2 (SAS Institute Inc., Cary, NC, USA) and R version 2.5.1 (R Project for Statistical Computing, Vienna, Austria).

## Results

Thirty-one of the 756 children in the study (~ 4%) had missing genotype information. Of the 725 children (53% male and 47% female) we genotyped, 74 (10.2%) were homozygous and 293 (40.4%) were heterozygous for the variant *DRD2* Taq IA allele (A1). The distribution of the variant allele was in accordance with the Hardy–Weinberg equilibrium (χ^2^ = 1.4821; *p*-value > 0.05).

The prevalence of anemia (hemoglobin < 11 g/dL) was 25%. The mean (± SD) blood lead, hemoglobin, and IQ, respectively, were 11.5 ± 5.3 μg/dL (range, 2.6–40.5 μg/dL), 11.8 ± 1.2 g/dL (range, 5.2–16.9 g/dL), and 107 ± 17 points (range, 42–166 points), and we found no significant differences by *DRD2* Taq IA genotype. Children who were homozygous variant had a higher number of siblings (*p*-value = 0.04), but other factors did not vary between homozygous variant and combined heterozygous and homozygous wild-type genotypes (Table 1).

After removal of outliers (lead and IQ: *n* = 8), 717 children remained in the study. The adjusted multivariate main effects GEE model (Table 2, model 1) indicated an inverse association between lead and IQ [−4.22 points; 95% confidence interval (CI), −7.10 to −1.36; for each one-unit increase in log blood lead (micrograms per deciliter)] and a positive association between hemoglobin and IQ (2.30 points; 95% CI, 1.26–3.08; for a 1-g/dL increase in hemoglobin). We observed no significant interaction between lead and hemoglobin (*p* = 0.39). *DRD2* Taq IA genotype was not a significant predictor of IQ. Children carrying the homozygous and heterozygous variant alleles had IQs 0.87 points lower (95% CI, −4.3576 to 2.6109) and 0.13 points lower (95% CI, −2.2122 to 1.9437), respectively, than children who were homozygous for the wild-type *DRD2* Taq IA allele.

In the models stratified by three-category genotype (Table 2, model 2), the inverse association between lead and IQ was stronger among children who carried the homozygous variant genotype than among children who carried the heterozygous or homozygous wild-type genotype. Increasing hemoglobin levels were associated with increasing IQ points among the children who were heterozygous (β estimate = 2.24; 95% CI, 0.57–3.91) and homozygous (β estimate = 3.03; 95% CI, 1.60–4.45) for the wild-type allele but was inversely associated with IQ among those homozygous for the variant allele (β estimate = −1.82; 95% CI, −5.28 to 1.64). Also, the magnitude and direction of the associations between lead and IQ and between hemoglobin and IQ were similar in the children who were heterozygous and homozygous wild-type for the *DRD2* Taq IA polymorphisms.

This suggested a recessive expression of the genotype, which we modeled by creating categories of *DRD2* Taq IA variant homozygous (A1/A1, *n* = 74) versus not Taq IA variant homozygous (carrying the wild type: A1/A2 + A2/A2; *n* = 651).

In the subpopulation carrying the *DRD2* Taq IA variant homozygote genotype (model 3), a unit increase in log blood lead (micrograms per deciliter) resulted in a decrease of 9.1 IQ points (95% CI, −18.08 to −0.16) compared with a decrease of 4.0 IQ points (95% CI, −7.21 to −0.69) among those who carried the wild-type genotype even after controlling for all other covariates. However, this difference in estimated effects of lead on IQ across the different *DRD2* Taq IA categories was not statistically significant (*p* > 0.1) in the interaction model (model 4), when modeled with and without the hemoglobin × *DRD2* term.

In contrast, the association between hemoglobin and IQ was consistently different in the *DRD2* polymorphism subgroups and in the interaction model. Higher hemoglobin was associated with an increase of 2.7 points of IQ among children with the wild-type alleles and was significant even after controlling for lead, midarm circumference, age, sex, parental education, family income, and number of children in the family unit (*p* < 0.0001). Among the homozygous variant children, a unit increase in hemoglobin was associated with a decrease of 1.8 points in IQ (i.e., the effect changed direction), but this was not statistically significant in this stratum (model 3). From the interaction model including the cross product term (hemoglobin × homozygous variant), a unit increase in hemoglobin was associated with 1.3 IQ points decrease (*p* = 0.02), after controlling for other variables (model 4). The shape of the dose–response relationship between hemoglobin and IQ in the different *DRD2* Taq IA genotype categories was linear.

## Discussion

Higher hemoglobin was associated with higher IQ in our study population and, in particular, among those children who are carriers of the wild-type allele (A2) of the *DRD2* Taq IA polymorphism. This is consistent with the findings from other studies. A meta-analysis of studies from Europe, North and South America, and the Middle East reported that a decrease of 1 g/dL hemoglobin was associated with a decrease in 1.73 IQ points (95% CI, 1.04–2.41) (Stoltzfus et al. 2007). Our finding of a decrease in 2.30 IQ points per 1-g/dL decrease of hemoglobin is consistent with the range of effect estimates reported.

However, this association did not hold among homozygous variant (A1) children, for whom increasing hemoglobin levels were associated with a decrease of 1.82 IQ points. This represents a reversal of the effect across the polymorphism and suggests that this genetic subgroup may have less potential for neuroprotective effects by iron supplementation.

Importantly, this points toward the role *DRD2* Taq IA polymorphism plays in modifying the effect of hemoglobin on cognition. In animal studies, iron levels are associated with changes in the dopamine system (Beard and Connor 2003; Unger et al. 2007). A study in iron-deficient rodents by Erikson et al. (2000) found that dopamine D2 receptor density in the nucleus accumbens and the ventral midbrain was reduced and associated with decreased D2 receptor–mediated activity. In their model of iron deficiency, they noted a decrease in the dopamine transporter (DAT) functioning, both *in vitro* and *in vivo*. They also detected a positive correlation between DRD2 and DAT density and hypothesized that the iron deficiency–induced reduction in DRD2 presynaptic receptors, which are involved in negative-feedback regulation of the dopamine system through increased DAT density and clearance of dopamine from the synapse (Dickinson et al. 1999; Meiergerd et al. 1993) and therefore may be responsible for the reduced DAT activity and density. Thus, decreased dopamine D2 receptor density along with iron deficiency, as noted by other studies (e.g., Youdim et al. 1989) may affect this regulatory pathway and affect dopamine neural signaling and associated behavior and cognition.

The mild adverse effect noted among homozygous variants could reflect a true adverse effect of hemoglobin on IQ; however, it may be an artifact due to the small number of children in this group, as evidenced by the wide variability in the effect estimate.

Even though we did not see a significant interaction between lead and *DRD2* genotype, the steep decrease in IQ associated with increasing blood lead among the children homozygous for the variant in the *DRD2* Taq IA allele suggests that these children may be more susceptible to the effects of lead on cognition. This is consistent with previous studies on lead and dopamine neurotransmission. A recent study by Froehlich et al (2007) suggests that the effect of lead on executive function among children is modified by a variable number of tandem repeats in the dopamine D4 receptor gene, indicating that polymorphisms in the dopamine system may be important sources of variability in the association between lead and childhood cognition.

In animal studies, lead has been shown to alter dopamine release, turnover, and receptor density (Cory-Slechta 1995, 1996, 1997; Zuch et al. 1998). Lead treatment is reported to cause differential and/or opposing effects on the nigrostriatal and mesolimbic dopamine systems. A study by Govoni et al. (1986) reported increased D2 binding in striatum but decreased binding in nucleus accumbens after lead exposure. However, in an autoradiographic study, chronic postweaning lead exposure was associated with decreased D1, D2, and DAT binding in the nucleus accumbens but not in the striatum (Pokora et al. 1996).

Much of the evidence of the role of the dopamine system in lead-induced neurocognitive impairments comes from animal studies that used fixed-interval schedule controlled behavior protocols, in which animals were repeatedly rewarded following scheduled time intervals of varying length for a specific behavior. In children ranging from 3 months to 9 years of age, response rates on fixed-interval schedules have been asserted to be a marker for impulsivity (Darcheville et al. 1993).

Environmentally relevant exposure levels of lead have repeatedly been shown to increase fixed-interval response rates, in some studies as much as 300% more than rates in control animals (Cory-Slechta et al. 1998, 2002). This may be attributable to increased nucleus accumbens dopamine levels, which are associated with increased fixed-interval responses. Mice exposed to lead and then administered dopamine agonists and antagonists showed markedly different fixed-interval responses. The study also suggested that lead-exposed mice had lower dopamine receptor (including DRD2) production, as demonstrated by the difference in recovery rates after being exposed to an irreversible antagonist (Cory-Slechta et al. 1998). There is evidence of enhanced sensitivity of D2 autoreceptors to alterations by lead (Zuch et al. 1998). This lends credence to the hypothesis that lead alters autoregulation of the dopamine system through DAT and presynaptic D2 receptors, which cause changes in baseline extracellular dopamine levels and downregulation of postsynaptic dopamine receptors (Cory-Slechta 1995).

Although there is considerable variation in the reports of how lead affects the dopaminergic system in different areas of the brain, there seems to be sufficient information to say that lead alters dopamine neurotransmission and that autoregulatory D2 receptors play an important role. Thus, it is biologically plausible that the findings of our study reflect the effect of lead on dopamine, which in turn regulates behavior and cognition.

Ours is the first epidemiological study to examine the role of a dopamine D2 receptor polymorphism in relation to the effects of lead exposure and hemoglobin on IQ and is also the first study to document the distribution of the *DRD2* Taq IA alleles in an Indian population. Although we studied 717 children, it is possible that the study was still underpowered to detect modification of the effect of lead on IQ, because the subpopulation of homozygous variant children accounted for only 10% of the population (*n* = 73). Further studies with larger sample sizes may be needed to determine if such a gene–environment interaction exists.

This study has several limitations. The cross-sectional observational design does not allow us to determine temporal or causal associations between lead/hemoglobin and cognition. The modification of these effects by genotype is, however, unaffected by the design. We also note that for both lead and hemoglobin, multiple prospective studies have demonstrated that exposures (high lead and low iron status) precede the decrease in neurological functions tested, and these associations are widely accepted.

We did not assess the quantity or quality of emotional and cognitive stimulation in the home environment using an instrument, such as the Home Observation for Measurement of the Environment Inventory, because a similar instrument that is culturally appropriate for the Indian subcontext does not exist. However, we did control for socioeconomic status (income, family size) and parental education, which have been shown to be moderately correlated with the stimulation provided to a child in the home (Seifer 2001).

The instruments used to measure blood lead and hemoglobin levels have been documented to have good field accuracy and precision, reducing the likelihood that substantial measurement error is present. Selection bias in this study is unlikely because 93% of the enrolled subjects participated in the full study and, moreover, comparison of the descriptive characteristics of those who provided blood samples and those who did not did not show any marked differences.

The findings of this study have important implications for developing countries, such as India, which have a high burden of anemia and elevated lead exposure among children. The most recent National Family Health Survey (2005–2006) estimated that 79% of all children 6–35 months of age in India are anemic (IIPS and Macro International 2007). In 1998–1999, approximately 45% and 50% of children < 3 years old in Delhi and Mumbai, respectively, had blood lead levels > 10 μg/dL (which is the level of concern by U.S. Centers for Disease Control and Prevention 1997) (Jain and Hu 2006). Lead was phased out of gasoline in India in 2000; however, childhood lead exposures continue to be elevated: Mean blood lead levels recently reported in children from urban areas range from 7.4 to 18.0 μg/dL (Ahamed et al. 2005, 2007; Nichani et al. 2006; Zimmermann et al. 2006). Our findings suggest that within such populations, children with the *DRD2* Taq IA genetic polymorphism may be bearing the greater burden of lead-associated IQ impairment. More important, they also may not benefit and, in fact, may suffer adverse effects from iron supplementation. Hence, the findings of this study are provocative and warrant further research.

## Figures and Tables

**Table 1:** Child and family characteristics by *DRD2* genotype.

Variable	Taq A1/A1 (*n* = 74)	Taq A1/A2 + A2/A2 (*n* = 651)
Child characteristics (mean ± SD)		

IQ	106.62 ± 17.34	107.32 ± 16.91
Blood lead (μg/dL)	11.66 ± 5.11	11.42 ± 5.43
Hemoglobin (g/dL)	11.95 ± 1.14	11.91 ± 1.19
Midarm circumference (cm)	16 ± 1.61	16.05 ± 1.48
Age (years)	5.05 ± 0.95	4.95 ± 0.93

Family characteristics [*n* (%)]		

Average monthly income (Indian rupees)		
< 2,000	12 (16.22)	97 (14.9)
2,000–4,000	30 (40.54)	291 (44.7)
4,000–6,500	19 (25.68)	157 (24.12)
> 6,500	13 (17.57)	106 (16.28)
Mother’s education level		
Illiterate/primary school	15 (20.27)	118 (18.13)
Middle school completion	23 (31.08)	224 (34.41)
High school certificate	22 (29.73)	152 (23.35)
College	14 (18.92)	157 (24.12)
Father’s education level		
Illiterate/primary school	11 (14.86)	76 (11.67)
Middle school completion	16 (21.62)	187 (28.73)
High school certificate	22 (29.73)	184 (28.26)
College	25 (33.78)	204 (31.34)
No. of other children		
0	12 (16.22)	148 (22.89)
1	47 (63.51)	419 (64.36)
≥ 2	15 (20.27)	84 (12.90)

**Table 2:** Effect of lead and hemoglobin on IQ by *DRD2* Taq IA genotype: results from stratified and interaction multivariate GEE models.^a^

			Lead [ln (μg/dL)]		Hemoglobin (g/dL)	
Model	Genotype	*n*	β-Coefficient (95% CI)	*p*-Value	β-Coefficient (95% CI)	*p*-Value
			−4.22 (−7.10 to −1.36)	0.004	2.30 (1.26 to 3.08)	< 0.0001
2: Stratified model	A1/A1	73	−9.12 (−18.08 to −0.16)	0.047	−1.82 (−5.28 to 1.64)	0.306
	A1/A2	290	−4.35 (−8.24 to −0.44)	0.023	2.24 (0.57 to 3.91)	0.001
	A2/A2	354	−3.87 (−8.28 to 0.54)	0.009	3.03 (1.60 to 4.45)	< 0.0001
3: Stratified model	A1/A1	73	−9.12 (−18.08 to −0.16)	0.046	−1.82 (−5.28 to 1.64)	0.302
	A1/A2 + A2/A2	644	−4.00 (−7.21 to −0.69)	0.017	2.72 (1.58 to 3.86)	< 0.0001
4: Interaction model^b^	A1/A1	717	−7.27 (−17.53 to −0.20)	> 0.28^c^	−1.32 (−4.66 to 2.02)	0.022^c^
	A1/A2 + A2/A2		−3.94 (−7.10 to −0.79)		2.72 (1.60 to 3.84)	

a Controlling for midarm circumference, age, sex, family income, parental education, and family size and accounting for school and classroom levels.

b The model assessing the interaction of lead and *DRD2* Taq IA controlled for confounding by hemoglobin and vice versa for the model assessing hemoglobin and *DRD2* Taq IA interaction.

c *p*-Values for interaction term coefficients.
